# Cognitive Gain or Handicap: Magical Ideation and Self-Absorption in Clinical and Non-clinical Participants

**DOI:** 10.3389/fpsyg.2021.613074

**Published:** 2021-02-26

**Authors:** János Kállai, Gábor Vincze, Imre András Török, Rita Hargitai, Sándor Rózsa, István Hartung, István Tamás, András Láng, Róbert Herold

**Affiliations:** ^1^Department of Behavioural Sciences, Medical School, University of Pécs, Pécs, Hungary; ^2^Pándy Kálmán Division, Department of Psychiatry, Békés Country Hospital Center, Gyula, Hungary; ^3^Institute of Psychology, Pázmány Péter Catholic University, Budapest, Hungary; ^4^Department of Psychiatry, Washington University School of Medicine in St. Louis, St. Louis, MO, United States; ^5^Institute of Psychology, Faculty of Humanities, University of Pécs, Pécs, Hungary; ^6^Department of Psychiatry and Psychotherapy, Medical School, University of Pécs, Pécs, Hungary

**Keywords:** absorptive capability, magical thinking, self-boundaries, schizophrenia spectrum disorders, socio-cognitive adaptation, mood disorders, anxiety disorders

## Abstract

**Background:** This study aimed to examine magical ideation and absorption traits across non-clinical and clinical groups to determine their potential adaptive and maladaptive functions.

**Method:** We enrolled 760 healthy participants from neighboring communities (female = 53.2%). Moreover, we recruited 318 patients (female = 66.5%), which included 25, 183, and 110 patients with schizophrenia spectrum disorders, anxiety disorders, and mood disorders, respectively. Potentially adaptive and maladaptive sociocognitive functions were measured to determine the role of magical ideation and self-absorption in patients with psychiatric disorders.

**Results:** The degree of magical ideation and absorption gradually increased in the following order: anxiety disorders, mood disorders, and schizophrenia spectrum disorders. Furthermore, enhanced self-absorption-related enhanced consciousness traits were essential indicators of the presence of self-integration weakness in patients with schizophrenia spectrum disorders.

**Conclusion:** Magical ideation and psychological absorption may be considered as mental model construction functions, which result in both gains and handicaps in social adaptation.

## Introduction

Magical ideation (MI) is considered a significant regressive cognitive process in several psychopathologies (Eckblad and Chapman, 1983; Claridge, 1997; Hanssen et al., 2003), somatoform disorders (Hausteiner-Wiehle and Sokollu, 2011), schizophrenia spectrum disorders (SSD), and neuropathologies (Wildt and Schultz-Venrath, 2004; Kelleher et al., 2012; García-Montes et al., 2014). However, in subclinical and healthy populations, MI facilitates creativity, interest in perceiving unusual experiences, and deep engagement in aesthetic sensations; moreover, it is associated with self-absorption (AB) (Mills and Lynn, 2000; Badzakova-Trajkov et al., 2011; Polner et al., 2018). Pathological MI is associated with SSD (Fonseca-Pedrero et al., 2014). MI onset occurs during early-childhood cognitive development, emerges from the preoperational thinking stage that appears in certain conditions, and remains active throughout adulthood (Miller and Ellen-Miller, 1989). MI is a developmental origin and meaning-making cognitive mechanism involving the perception of a loss of control of executive function and logical supervision over personal sensations of reality. It is a belief set that is associated with supernatural experiences, including telepathy, clairvoyance, and a drive to step into mindfulness meditation and altered conscious states, which are considered self-integration endeavors (Antonova et al., 2016). The cognitive mechanism underlying MI is closely associated with the cognitive symptoms of schizotypy (Cicero et al., 2015), as well as the cognitive processing of unusual events, which involve absorbed, weak, and less-defined self-boundaries (Rosen et al., 2017).

AB is a self-integrity disorder that is associated with regressive cognitive function and facilitates social adaptation and attachment; however, it may also be a cognitive risk for developing mental disorders. The self-reported absorption capability refers to a disposition to shift perceptual and experiential methods upon entering a private world and interpreting the conventional physical and social environments through an individual experience-driven perspective. AB is associated with vivid imagery capability, synesthetic perceptions, daydreaming, openness toward unusual experiences, (Glisky and Kihlstrom, 1993), altered attention function, and a willingness to transform conventional cognitive and perceptual processing modes (Kremen and Block, 2002). Similar to MI, AB involves a regressive set of presentations that are inherent to regressive cognition, as well as creative productions in patients with psychopathological disorders and healthy individuals (Glicksohn and Barrett, 2003; Perone-Garcelán et al., 2013; Humpston et al., 2016). AB is associated with vivid fantasy, decreased self-awareness, and increased alterations in consciousness states (Pekala et al., 1985). Furthermore, AB is involved in experience, creativity, and the manifestation of flow experiences; additionally, it significantly affects the severity of psychopathological symptoms (Wild et al., 1995; Léger et al., 2014).

### Maladaptive Behavior: Social Withdrawal and Schizotypy

Sociocognitive maladaptation is characterized by fearful withdrawal from social relationships, incompetency feelings, and limited agency in social problem-solving. This avoidance behavior appears in normal persons, as well as among individuals with higher scores in phobias and anxiety disorders. Social avoidance has been associated with regressive cognitive functions, elevated perceptual subjectivity, and certainty in construct boundaries among conceptual categories (Barlow, 2002; Kállai et al., 2007; Blanchard and Blanchard, 2008). The healthy endpoint of SSD might present moderate symptoms, which involves low scores in the schizotypal personality traits; contrastingly, the pathological endpoint involves an increased vulnerability to schizophrenia and schizoaffective disorders (Barrentes-Vidal et al., 2015). The maladaptive rate shows a tendency from schizotypy to schizophrenia (Debbané and Barrantes-Vidal, 2015). The Fear Survey Schedule and Schizotypy Personality Questionnaire is a self-reported assessment tool for measuring different maladaptation facets.

### Sociocognitive Adaptation: Self-Concept Clarity and Self-Esteem

Adaptive regulation for prosocial behavior involves manipulating self-boundaries and certain responsiveness to another individual, as well as attentional shifts between oneself and another person's mental state. This dynamic shifting between the first- and third-person perspectives is dependent on the degree of self-concept coherence (Bigler et al., 2001; Carleton et al., 2010; Kállai et al., 2019). However, the integration between cognitive and affective domains in self-related experiences remains unclear (Thakkar and Park, 2010; Healey and Grossman, 2018). Affective functions are associated with the degree of self-disturbances and positively affect self-coherence construction (Mishara et al., 2014). Contrastingly, cognitive functions are focused on constructing a clear reference frameshifting between self-related and self-independent environmental object-oriented representations (de Vignemont, 2018). There are different ways of measuring the discrimination rate between oneself and another person/object. One such method involves the clarity of self-definition revealing the self-coherence degree, which is a basic condition for socio-cognitive adaptation. A blurred self-concept is related to anxiety, fears, and SSD; further, it is considered a maladaptive functional deficit (Ritchie et al., 2011; Cicero, 2017). A high score in self-concept clarity is associated with several adaptive traits, including self-esteem. Contrastingly, low scores in self-concept clarity, as well as dysregulation of identity and self-direction, are associated with anxiety, behavioral avoidance (Kusec et al., 2016), loneliness (Frijns and Finkenauer, 2009), and personality disorders such as schizotypy (Roepke et al., 2013; Pincus et al., 2019). Schizotypy is a multidimensional construct (e.g., cognitive, affective, and disorganized domains) that covers symptoms ranging from normal to schizophrenia. Self-esteem and self-concept clarity could be relevant adaptive traits for measurements in both clinical and non-clinical participants. The Self-Concept Clarity Scale and the Rosenberg Self-Esteem Scale may be appropriate for assessing these traits.

### Hypothesis

This study aimed to assess non-clinical and clinical participants to determine the intensity rate of MI and AB personality traits and pathologies. This study sought to explore the maladaptive effects of MI and AB based on social anxiety, fear-related avoidance, and schizotypal traits, which are involved in the maladaptive social withdrawal from social relationships. Based on recent studies (Sass and Parnas, 2003; Kwapil et al., 2013; Postmes et al., 2014), schizotypal traits are considered as neurodevelopmental self-integration disorders. Psychological vulnerability for schizophrenia ranges from normal adaptation to maladaptation. To determine the origin of the adaptive and maladaptive nature of the aforementioned self-integration difficulties, this study sought to measure the MI intensity and the predispositions of absorption psychological traits. Contrastingly, self-esteem and self-concept clarity scales were used to assess the adaptive role of MI and AB. This could elucidate new elements in the adaptive/maladaptive trait constellations in both healthy individuals and psychopathology cases. Specifically, this study aimed to reveal the role of MI and AB in sociocognitive adaptation in both healthy participants and patients with several psychopathologies. This study sought to examine patients with SSD (*Clinical SSD*), anxiety disorders (*Clinical A*), and mood disorders (*Clinical M*), as well as non-clinical healthy *(HE*) participants.

Given that diagnostic classifications are yet to directly reflect the degree of acute sociocognitive adaptation, this study purposed to use personality trait measures to identify the rate of social adaptation and maladaptation. This study sought to elucidate the differences in the degree of MI and AB between clinical and non-clinical cases. Given that MI and AB are strongly associated with the intensity of psychopathological symptoms (Wild et al., 1995; Fonseca-Pedrero et al., 2014), there could be between-group differences in the contribution of the several AB factors to the symptom intensity rate. MI and AB are considered as manifestations of self-integrity weakness, which could be involved in social maladaptation, especially in patients with SSD. We proposed that the maladaptive effects of MI and AB are synergistic and indicative of the maladaptation degree in anxiety disorders, mood disorders, and SSDs.

## Materials and Methods

### Participants and Procedure

This study examined 1,103 individuals using a questionnaire packet. Subsequently, 25 individuals were excluded due to incomplete test administration. Consequently, the final sample was comprised of 1,078 individuals. The clinical sample contained outpatients with anxiety disorders, mood disorders, or SSD who were enrolled from several psychiatry departments between September 2017 and September 2018. The clinical sample comprised 318 individuals [238 women (66.5%, mean age = 45.3 years; SD = 12.6) and 80 men (33.5%, mean age = 42.9 years; SD = 11.6)] with an age range of 18–65 years. The non-clinical sample comprised 760 individuals recruited from neighboring communities [493 women (53.2%, mean age = 38.2 years; SD = 9.5) and 267 men (46.8%, mean age = 35.8 years; SD = 10.9)] with an age range of 18–65 years. These individuals were enrolled in 3 different country regions. Supplementary Table 1 presents other demographical characteristics. Tables 1, 2 present the descriptive statistics and sex differences in the entire sample. The clinical sample comprised outpatient individuals with a history of acute treatment in psychiatric departments, regional country hospitals, and local psychiatric clinics. The inclusion criteria were patients with anxiety disorders, mood disorders, and SSD. We excluded patients with neurocognitive deficits or drug abuse. Syndromes were coded based on the International Classification of Disease version 10 (ICD-10-CM) manual, which describes anxiety disorders (F40–F48), SSD (F20–F29), and mood disorders (F30–F39). The diagnostic classification was based on the Diagnostic and Statistical Manual of Mental Disorders, 5th Edition (DSM-5) coding list for ICD-10-CM; moreover, it was confirmed by two professionals (JK and RH). Supplementary Table 2 presents the diagnostic characteristics of the recruited patients. Participation was voluntary; moreover, the participants did not incur costs for the examinations. The study was conducted following the Helsinki Declaration (ethical allowance No. 6732 PTE/2017).

**Table 1:** Descriptive statistics of adaptive and maladaptive personality trait predispositions, as well as decreased self-integration, in the entire sample.

**Adaptive/maladaptive trait predispositions**	***n***	**Mean (SD)**	**Median**	**Skewness**	**Kurtosis**	**Min–max**
**Adaptive**						
RSES	1,074	29.56 (6.5)	30	−0.4	−0.4	10–40
SCCS	1,078	44.42 (9.6)	47	−1.1	0.6	11–55
**Maladaptive**						
FSS	1,061	62.55 (33.9)	57	0.8	0.5	0–187
SPQ_BR	1,078	40.68 (18.7)	39	0.7	0.4	7–120
**Decreased self-awareness**						
MIS	917	8.107 (4.4)	7	0.9	1.0	0–27
TAS	1,076	89.42(25.8)	88	0.2	−0.4	34–168

*RSES, Rosenberg Self-Esteem Scale; SCCS, Self-concept Clarity Scale; FSS, Fear Survey Schedule; SPQ_BR, Schizotypal Personality Questionnaire-Brief Revised; MIS, Magical Ideation Scale; TAS, Tellegen Absorption Scale*.

**Table 2:** Sex differences in the entire sample.

**Self-Integration**	***n***	**Mean Male, Female (SD)**	***t*-values**
Self-esteem (RSES)	347	30.72 (5.6)	4.31^***^
	727	29.01 (6.8)	
Self-concept clarity	347	45.10 (9.1)	1.52
(SCCS)	731	44.17 (9.8)	
Self-related fear and	343	49.40 (28.3)	−9.71^***^
avoidance, (FSS)	718	68.80 (34.6)	
Self-boundary	347	39.11 (18.2)	−1.92
weakness (SPQ-BR)	731	41.42 (19.0)	
Magical ideation MIS	302	7.24 (4.3)	−4.06^***^
	615	8.48 (4.2)	
Self-absorption (TAS)	347	85.28 (24.4)	−3.73^***^
	729	91.39 (26.2)	

*** *p < 0.001*.

### Measurement

#### The Rosenberg Self-Esteem Scale

The Rosenberg Self-Esteem Scale (RSES) (Rosenberg, 1965; Sallay et al., 2014) is a unidimensional model for measuring adaptive self-functions, including self-confidence and self-depreciation traits. It contains 10 items that are answered based on a response scale ranging from strongly agree (4) to strongly disagree (1), along with reverse-scored items. High scores are indicative of high self-esteem, which is associated with better interpersonal adaptation (Ciarrochi et al., 2007) and mature self-regulation (Pyszczynski et al., 2004). In our sample, Cronbach's α was 0.91.

#### The Self-Concept Clarity Scale

The Self-Concept Clarity Scale (SCCS) (Campbell et al., 1996; Hargitai et al., 2020) is comprised of 12 items answered on a 5-point Likert scale, which ranges from strongly disagree (1) to strongly agree (5). The SCCS is a self-reported questionnaire for self-coherence measurement. A high score is indicative of high self-concept clarity, while a low score indicates a blurred self-concept and weakness in self-regulation (Cicero et al., 2015; de Sousa et al., 2016). In this study, this questionnaire had excellent internal consistency (Cronbach's α = 0.91).

#### The Fear Survey Schedule

The Fear Survey Schedule (FSS) (Arrindell et al., 2003) measures fearful behavioral avoidance when anticipating a conventional situation as harmful. It is comprised of 52 items for rating fearfulness in several situations. All the items are answered on a 5-point Likert scale ranging from 0 to 4. Behavioral and cognitive avoidance has been associated with anxiety, as well as social and physical maladaptation; further, it is crucially involved in the severity of normal to clinically relevant symptoms. In this study, a high total FSS score was indicative of an intensive avoidance bias to anxiety disorders. Cronbach's alpha was excellent (α = 0.95).

#### The Schizotypal Personality Questionnaire-Brief Revisited

The Schizotypal Personality Questionnaire-Brief Revisited (SPQ-BR) (Cohen et al., 2010; Kállai et al., 2018) is a tool for assessing individual vulnerability to schizotypy on a 5-point Likert-style scale ranging from 0 (strongly disagree) to 4 (strongly agree). Higher scores are reflective of greater social and behavioral maladaptation, as well as a higher SSD risk. The SPQ-BR is comprised of 32 items classifiable as Cognitive, Interpersonal, and Disorganized main factors. In this study, the total SPQ-BR score was indicative of the degree of sociocognitive maladaptation. Cronbach's alpha was excellent (α = 0.91).

#### The Magical Ideation Scale

The Magical Ideation Scale (MIS) was introduced by Eckblad and Chapman (1983) and contains 30 items that are scored based on a true/false response format (national adaptation by Rózsa et al., 2020). The MIS is a unidimensional scale for measuring the rate of unrealistic invalid causations and false beliefs, as well as assessing schizotypal traits and proneness to psychosis (a few sample items: “If reincarnation were true it would explain some unusual experiences I have had” and “I have worried that people on other planets may be influencing what happens on Earth”). A higher MIS score is closely associated with perceptual aberration, physical anhedonia, and psychoticism. In both teenagers and adults, a higher MIS score is indicative of more pronounced magical thinking. In our study, the internal consistency was good (Cronbach's α = 0.77).

#### The Tellegen Absorption Scale

The Tellegen Absorption Scale (TAS) (Tellegen and Atkinson, 1974; Simor et al., 2011; Rózsa et al., 2019) is comprised of 34 items that are ordered based on six factors. All items are answered on a 5-point Likert scale ranging from 0 (strongly disagree) to 4 (strongly agree). This scoring method has been previously applied (Carleton et al., 2010). The scale has the following six subscales: Responsiveness to Engaging Stimuli (RES), Synesthesia (SY), Enhanced Cognition (EC), Oblivious/Dissociative Involvement (DI), Vivid Reminiscence (VR), and Enhanced Awareness (EA) (example items: “Sometimes I feel someone is present even though they are not physically present” and “The sundown has an intensive impression on my mind”). In this study, the internal consistency of the total AB scale score was excellent (Cronbach's α = 0.94). The consistency indexes of the subscales were adequate: RES, 0.80; SY, 0.84; EC, 0.83; DI, 0.82; VR, 0.65; and EA, 0.74.

### Data Analysis

Statistical analyses were performed using IBM SPSS for Windows 22.0. Although the Kolmogorov–Smirnov test revealed significant deviation from normality for all variables, skewness and kurtosis of all variables laid within the range of |2.0| that indicated normal distributions for all variables (Kim, 2013). Therefore, we used parametric tests. Sex differences were analyzed using *t*-tests. Between-variable associations were tested using partial correlation with adjustment for age and sex. Between-group differences in measured variables were determined using multivariate analysis of variance (MANOVA) with Tukey's honestly significant differences (Tukey's HSD) as *post-hoc* tests.

## Results

There were sex differences in all the measured adaptive and maladaptive traits except for self-concept clarity and schizotypy scores (Table 2). Female individuals showed higher scores in fearful avoidance, MI scores, and AB scores. Contrastingly, male individuals showed higher scores in self-esteem. Although determining sex differences were not the main study objective, it was considered a potential covariate in subsequent analysis.

The SBQ-BR contains summarized scores of cognitive, interpersonal, and disorganized behavior sub-factors of schizotypy. The TAS is comprised of sub-scales that yield scores of the responsiveness to engaging stimuli, synesthesia, enhanced cognition, dissociative involvement, vivid reminiscence, and enhanced awareness scales.

The correlation matrix for the entire sample shown in Table 3 revealed an association of MI and AB with both adaptive and maladaptive personality traits; however, there were pattern differences. High scores in the MI and TAS were associated with low scores on adaptive trait predispositions (RSES and SCCS) and high scores on maladaptive traits (FSS and SPQ-BR) concerning self-integration capabilities. However, there remained several critical areas. The social avoidance and schizotypy-linked self-deficits have emerged in most mental disorders; furthermore, MI and AB are manifested in different mental disorders as well but differ in their intensity.

**Table 3:** Sex- and age-controlled partial correlations between MI, absorption capability, and adaptive (RSES, SCCS) and maladaptive (FSS, SPQ_BR) traits in the entire sample.

**Variables/sex and age**	**2**	**3**	**4**	**5**	**6**
1. RSES	0.606^**^	−0.439^**^	−0.569^**^	−0.214^**^	−0.079^*^
2. SCCS_	–	−0.483^**^	−0.668^**^	−0.401^**^	−0.318^**^
3. FSS		–	0.491^**^	0.268^**^	0.187^**^
4. SPQ BR			–	0.521^**^	0.402^**^
5. MIS				–	0.540^**^
6. TAS					–

* *p < 0.5*;

** *p < 0.01*.

*RSES, Rosenberg Self-Esteem Scale; SCCS, Self-Concept Clarity Scale; FSS, Fear Survey Schedule; SPQ-BR, Schizotypal Personality Questionnaire-Brief Revised; MIS, Magical Ideation Scale; TAS, Tellegen Absorption Scale*.

To assess the effect of sex and diagnostic classification on adaptive (RSES, SCCS) and maladaptive (FSS, SPQ_BR, MIS, TAS) traits, we employed a 2 (female vs. male) × 4 (Clinical A vs. Clinical M vs. Clinical SSD vs. HE) MANOVA. The test did not reveal multivariate [(Wilk's λ = 0.981; F_(18, 2526.271)_ = 0.975; *p* = 0.486) or univariate all [F_(3)_s ≤ 2.220; all *p* ≥ 0.084)] effects of the sex × diagnostic classification on the dependent variables. Therefore, we conducted a one-way MANOVA to determine the effect of diagnostic classification on the measured variables without sex categorization. There was a significant multivariate effect of diagnostic classification on the measured variables [(Wilk's λ = 0.658; F_(18, 2537.584)_ = 22.452; *p* < 0.001)]. There were significant differences among diagnostic groups (Clinical A vs. Clinical M vs. Clinical SSD vs. HE) in the measured adaptive and maladaptive traits. Table 4 presents the results of the univariate analyses and *post-hoc* tests (Tukey's HSD).

**Table 4:** Comparison of the diagnostic groups on adaptive (RSES, SCCS) and maladaptive (SPQ-BR, MI, AB) traits.

**Self-** **regulation**	**Schizophrenia** **spectrum**	**Anxiety** **disorder**	**Mood** **disorder**	**Healthy**				
	**SSD**	**A**	**M**	**HE**	***F***	***p***	**Partial η**^**2**^	**Tukey's HSD**
	***n* = 21** **M(SD)**	***n* = 86** **M (SD)**	***n* = 84** **M (SD)**	***n* = 715** **M (SD)**				
RSES	23.90 (5.1)	26.59 (7.5)	23.31 (6.0)	31.45 (5.6)	69.298	<0.001	0.187	M, SSD < SSD, A < HE
SCCS	33.48 (10.6)	42.49 (10.8)	37.23 (11.0)	46.79 (7.8)	49.028	<0.001	0.140	SSD, M < A < HE
FSS	82.71 (31.2)	76.07 (40.6)	91.92 (41.4)	54.75 (26.9)	50.460	<0.001	0.144	HE < A, M, SSD
SPQ-BR	60.14 (19.2)	45.90 (22.0)	61.19 (21.9)	35.92 (14.9)	74.494	<0.001	0.199	HE < A < M, SSD
MIS (MI)	12.85 (5.67)	7.22 (3.8)	11.88 (5.3)	7.61 (4.0)	36.437	<0.001	0.108	A, HE < M, SSD
TAS (AB)	103.71 (23.5)	80.66 (23.2)	92.89 (26.9)	91.10 (25.5)	6.570	<0.001	0.021	A, HE, M < SSD

*SSD, schizophrenia spectrum disorders; A, Anxiety Disorders; M, Mood disorders; HE, Healthy*.

Compared with clinical groups, the non-clinical group showed significantly more positive self-esteem. In the clinical groups, patients with anxiety disorders had significantly greater positive self-esteem than those with mood disorders. There was no significant difference in self-esteem levels between patients with SSD and the other two clinical groups.

Regarding self-concept clarity, the non-clinical sample showed significantly higher self-concept clarity than the clinical sample. Concerning clinical groups, patients with anxiety disorders had clearer self-concept than patients with mood disorders or SSD, with the latter 2 groups showing no significant difference.

Regarding avoidance behavior in fear-provoking situations (FSS), the non-clinical sample showed significantly lower avoidance levels than the clinical sample. Moreover, participants with anxiety disorders showed significantly lower avoidance levels than patients with mood disorders.

Regarding schizotypal traits, there was no difference between patients with mood and SSD; however, these groups showed a greater presence of schizotypal traits (SPQ-BR) than that in patients with anxiety or healthy individuals. Furthermore, the SPQ-BR scores of patients with anxiety were significantly higher than those of the non-clinical sample.

For MI, patients with anxiety and healthy individuals showed significantly lesser MI than patients with mood disorders and SSD. Regarding AB, participants with anxiety disorders showed significantly lower AB levels than those with SSD. There was no other between-group difference in AB.

**Figure 1:**
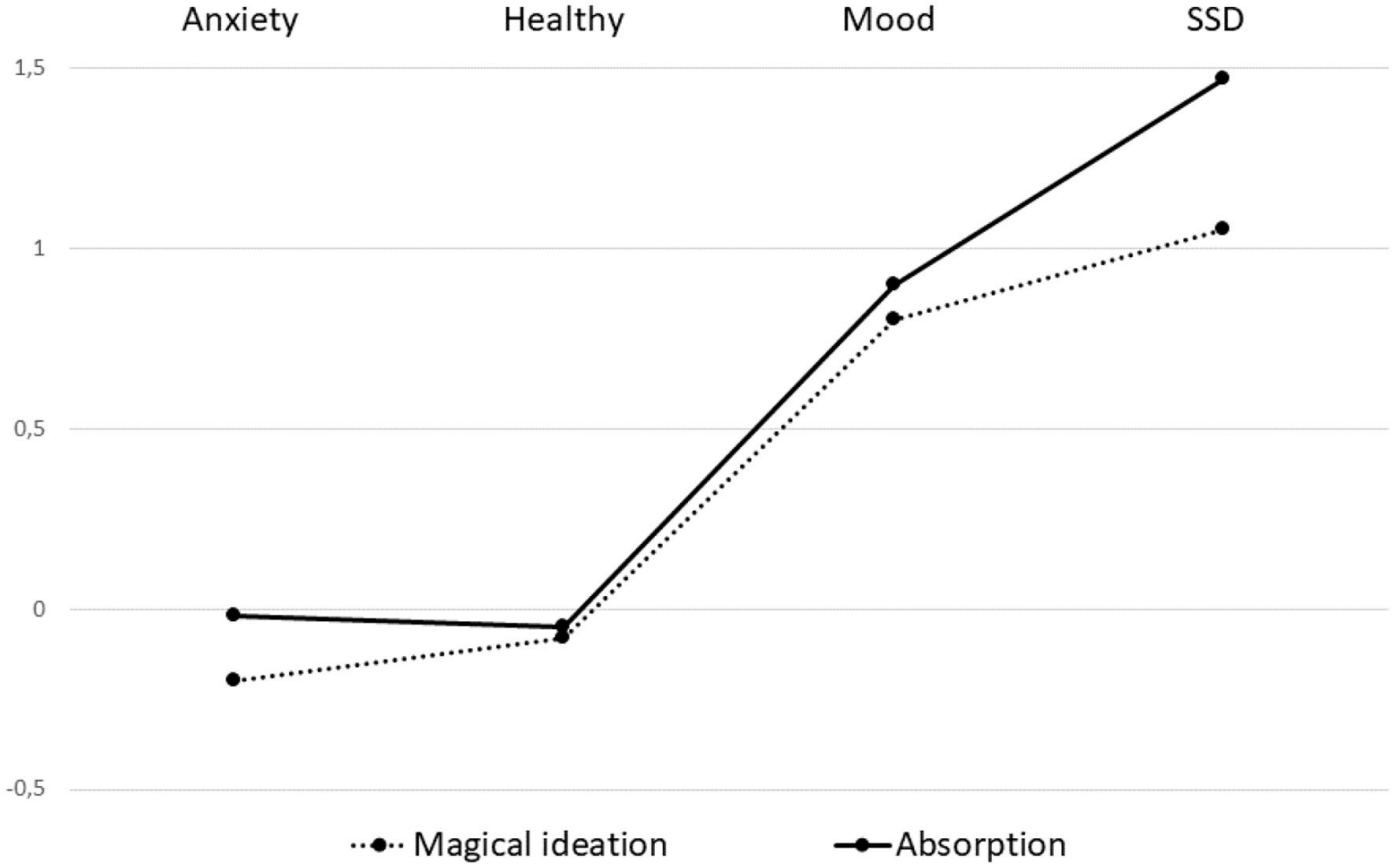
The degree of magical ideation (MI) and self-absorption (AB) in the patients with anxiety, mood, and schizophrenia spectrum disorders, as well as in the healthy control group.

The main study objective was determining the MI and AB predispositions and patterns in clinical and non-clinical samples. The MIS is a unidimensional construct, with its results being shown in Table 4. On the other hand, we performed a detailed analysis of the six TAS subscales (responsiveness to engaging stimuli, synesthesia, enhanced cognition, dissociative involvement, vivid reminiscence, and enhanced awareness) to reveal the representation of AB capabilities in both the clinical and non-clinical samples. Before analysis of the MI and AB rate in the two samples, the MI and AB total scores underwent standardization. As shown in Figure 1, except for in the non-clinical sample, there was a tendency of a gradual increase in the MI and AB in the following order: patients with anxiety disorders, patients with mood disorders, and patients with SSD.

One-way ANOVA was used to assess the different AB components in the clinical groups, which revealed significant among-group differences in all AB facets (Table 5). However, regarding the VR subscale, the *post-hoc* analysis revealed that all diagnostic groups formed one homogeneous subset, i.e., there was no among-group HSD. For the RES, SY, DI, and EA subscales, patients with anxiety disorder showed the lowest scores, which were significantly lower than those of patients with SSD. However, there was no significant difference between the aforementioned groups and patients with mood disorders and healthy individuals.

**Table 5:** The absorption subscales in different groups of patients and healthy participants.

**TAS-subscales**	**Schizophrenia spectrum**	**Anxiety disorder**	**Mood disorder**	**Healthy**			**Partialη**^**2**^	**Tukey's HSD**
	**SSD**	**A**	**M**	**HE**	***F***	***p***		
	***n* = 25** **M (SD)**	***n* = 182** **M (SD)**	**n = 110** **M (SD)**	***n* = 759** **M (SD)**				
RES	3.16 (0.7)	2.62 (0.9)	2.92 (1.0)	2.94 (0.8)	7.88	<0.001	0.022	A, M, HE < M, SSD
SY	2.82 (1.0)	2.19 (0.8)	2.50 (0.9)	2.54 (0.9)	8.91	<0.001	0.024	A, M, HE < M, SSD
EC	2.77 (0.8)	2.27 (0.8)	2.66 (0.9)	2.56 (0.9)	7.32	<0.001	0.020	A, HE < M, SSD
DI	3.11 (0.8)	2.49 (0.9)	2.79 (1.0)	2.83 (0.9)	7.57	<0.001	0.021	A, M, HE < M, SSD
VR	2.92 (1.0)	2.55 (0.9)	2.76 (0.9)	2.75 (0.9)	3.09	<0.034	0.008	OHS
EA	2.91 (1.0)	2.24 (0.8)	2.59 (0.9)	2.29 (0.8)	8.79	<0.001	0.024	A, HE, M < M, SSD

*RES, the responsiveness to engaged stimuli; SY, synesthesia; EC, enhanced cognition; DI, dissociative involvement; VR, vivid reminiscence; EA, enhanced awareness; OHS, one homogeneous subset*.

Regarding the EC subscale, patients with anxiety disorder showed the lowest scores, which were significantly lower than those of the patients with mood disorders or SSD. However, there was no significant difference between the aforementioned groups and healthy individuals.

For the EA subscale, the patients with anxiety and healthy individuals showed significantly lower scores than the patients with SSD. There was no difference in the EA scores between patients with anxiety disorders and healthy individuals. Further, the scores of patients with mood disorders did not differ from those of the other clinical groups.

## Discussion

This study sought to determine the role of MI and AB in clinical and non-clinical samples. Both MI and AB fundamentally involve regressive cognitive strategies, which manifest as socially adaptive behavior or mental disorders with different intensity rates. MI is a constitutive agent of creative thinking, moreover meaning-making in a period in childhood, which is considered as a potential handicap to reality construction in adulthood. *First*, we investigated sex differences in the measured self-regulation traits and observed commonly reported differences, including higher self-esteem levels among male than among female individuals (Kling et al., 1999; Bleidorn et al., 2016). Furthermore, female individuals had higher levels of social fear, MI, and AB than male individuals (Tellegen and Atkinson, 1974; Arrindell et al., 2003; Karcher and Shean, 2012). For the entire sample, female individuals showed higher MI and AS scores than male individuals. *Second*, sex- and age-controlled partial correlation coefficients in the entire sample revealed a strong association between MI and AB; moreover, they had contrasting relationships with adaptive and maladaptive traits. Specifically, the MI and AS scores were higher in maladaptive and lower in adaptive trait predispositions. *Third*, the intensity of MI and AB in the clinical sample, but not in the non-clinical sample, gradually increased in the following order: patients with anxiety disorders, patients with mood disorders, and patients with SSD. There was no significant difference in the MI and AB intensity between healthy individuals and patients with anxiety disorders. However, healthy individuals showed greater adaptive trait predispositions (RESE and SCCS) than patients with anxiety disorders. *Fourth*, regarding the pattern of AB factors in the clinical sample, enhanced (expanded) awareness was crucially involved in self-integration disorders in patients with SSD and mood disorders. Further, patients with SSD presented with extremely high expanded awareness, dissociative involvement, and responsiveness to engaging stimuli. Contrastingly, healthy participants and patients with anxiety disorders showed a limited degree of expanded awareness. The findings of the lowest EA score in patients with anxiety disorders indicated a fixation to first-person-perspective representations. Moreover, there was a concomitantly restricted expansion toward the third-person perspective of the environment; on the other hand, patients with SSD showed a more pronounced expanded self-awareness. It seems they experience their environment as if it would be as a part of their own space and own body.

It has been reported that MI is associated with creativity, prosocial adjustment, and meaning-making function (Rosengren and French, 2013; Fink et al., 2014). However, we observed a negative correlation between MI with self-esteem and self-coherence. Therefore, a high MI could be crucially involved in both prosocial adaptation and instability in self-regulation. Moreover, it is associated with weak self-integration capabilities, as well as increased responsiveness to social fears and avoidance. Currently, this complex relationship remains unclear given that the self-reported MI measure has both adaptive and non-adaptive components. The MIS is composed of different elements, namely, paranormal beliefs and magical causality, which are on opposite ends of the magical dimension (Horan et al., 2004; Chun et al., 2019; Rózsa et al., 2020). Magical causality facilitates knowledge generation during unusual experiences. Specifically, the meaning-making process generates intuitive knowledge for interpreting uncertain situations; consequently, it acts as defense mechanisms and decreases concerns regarding unfamiliar situations (Wildt and Schultz-Venrath, 2004; Rosengren and French, 2013). Therefore, this is a culturally essential adaptive function. However, paranormal beliefs are associated with delusions and hallucinations, which are frequently found in maladaptive psychopathologies (García-Montes et al., 2014). MI reduces logical control over clear definitions of conceptual categories; additionally, it contributes to cognitive symptom manifestation in SSD, specifically in schizotypy.

Furthermore, in patients with SSD, AB is a strong predictor of the intensity of both positive and negative symptoms (Cicero et al., 2016). However, they are adaptive in cases where a person is immersed in social/physical reality or a computer-generated virtual environment (Léger et al., 2014). AB is involved in deconstructing the spatial and temporal context of cognition; further, it facilitates engagement in an unusual and wide range of sensory, spiritual, and dissociative experiences (Luhrmann, 2017). MI is a regressive cognitive function that constructs a subjective reality that differs from common-sense physical and social reality. MI is only helpful during a short developmental time window since it inhibits mental control maturation, which refers to the spatial and temporal organization of the common-sense base reality. Furthermore, MI limits long-term adaptation to adulthood requirements (Rosengren and French, 2013). This deficit in time perception impedes autobiographical memory and evaluation of the expected behavioral consequences (Berna et al., 2016; Brashier and Multhaup, 2017). The first phase of childhood cognitive development involves vast development for modeling and representation of feasible knowledge to understand reality. However, in adulthood, in the face of stress and complex challenges, MI changes occur across sexes and ages (Garzitto et al., 2016), which result from deficits in both social adaptation and executive functions (Karcher and Shean, 2012). Consequently, the early observed gains subsequently lead to deficits.

### MI and AB Concerning Differences Between Patients With SSD and Healthy Individuals, as Well as the Related Biological Mechanisms

As an endophenotype trait, schizotypy could persist through sex-related mechanisms (Grant et al., 2013) and play multiple roles in cognitive development, social selection, and adaptation. Although SSD is a maladaptive disorder, non-schizophrenic offspring of parents with schizophrenia often display adaptive behaviors, exhibit creative performances, maintain adequate peer relationships, and follow academic or artistic professions (Power et al., 2015). MI-related activity is heritable (Karcher et al., 2014) and manifests as creative performance, flow, and unusual experiences in adulthood (Mohr and Claridge, 2015; Polner et al., 2018). However, in patients with SSD, it primarily manifests as cognitive symptoms (Raynal et al., 2016). Taken together, MI could be considered a regressive cognitive function that yields both adaptive and maladaptive outcomes. This heritage manifests with brain structural variations. For example, lesions in the pre-frontal area of the brain, which is involved in controlling and regulating “fast” (non-logically controlled) and “slow” (logically controlled) cognitive processing, causes MI enhancement (Sloman, 2014; Zhong et al., 2018). Moreover, functional MRI (fMRI) studies on dual-process tasks reported differences in the activity in areas of the default mode network, which indicated that non-logically controlled MI was associated with activity in the medial pre-frontal cortex, posterior cingulate cortex, inferior parietal lobe, and lateral temporal cortex (Buckner et al., 2008; van Buuren et al., 2012). Additionally, structural MRI examinations have reported volume differences between participants with high and low MI activity in the right middle temporal cortex and left precuneus (Kapogiannis et al., 2009). Studies on agents for neurochemically facilitating delusions, MI-related psychedelic experiences, and self-transcendent experiences have shown that the 5-HT(A2) serotonin receptor–ligand plays a major role in the return of regressive superior pattern-processing functions (Borg et al., 2003; Mattson, 2014).

Our findings further confirm that MI has a biologically directed cognitive developmental trajectory, is associated with brain maturity (Dubal and Viaud-Delmon, 2008; Markle, 2010), and is a major function in the superior pattern-processing system for anticipating the meaning of unfamiliar stimuli patterns. The meaning-making function of magical thinking in non-clinical individuals is representative of an element of the schizotypal person's cognitive set. Furthermore, without adaptive self-regulation mechanisms, this function is considered a risk factor for hallucinations and abnormal perceptions. From a practical and therapeutic perspective, there is a potential role of AB and MI in the remediation of regressive cognitive functions facilitated by art therapy in patients with SSD. Our findings demonstrate the relevance of dance, movement, and creativity-based therapeutic rehabilitation programs, as suggested by Buttingsrud (2018), Colombetti (2014), and Legrand and Ravn (2009), which further provide outlets for self-embodied reflection. Therefore, there might be a need to focus on expanded awareness for improving self-integration skills when a patient is absorbed in dance and music experiences, as well as when they participate in thought and action coherence training.

## Limitations

This study has several limitations. First, there was a general female overrepresentation in the clinical sample. This sample asymmetry could also occur when examining psychological parameters in a healthy population. Furthermore, female individuals present with a higher MI, ability to absorptive sensations, and willingness to intake absorptive sensations. Although this limitation can be mitigated by sex-controlled statistical analysis, the asymmetry should be considered when reaching a conclusion and interpreting data. Second, compared with the other clinical groups, there was a small number of patients with SSD, which reduces the generalizability of the findings to the entire patient population. Future studies should confirm these findings with a larger group of patients with a different SSD type.

## Conclusion

Our findings indicated that MI and AB may be considered as mental model construction functions that result in handicaps and gains in social adaptation. On the one hand, there is a regressive mental function that constructs new meaning in an incompletely represented environment, which facilitates social adaptation, as well as creative scientific and art performances. AB and MI are considered to involve a prospective capacity to expend reality perception without checking the causal relationship or employing adequate executive control. These aforementioned functions are present in healthy intuitive thinking and adaptive behaviors. On the other hand, MI and AB are essentially involved in enhancing the intensity of psychopathology symptoms, especially in cases with self-integration disturbance. Moreover, they represent an attentional function that oscillates between the first- and third-person view models of the constructed reality. Art, dance, movement, and physical nature-based therapies allow individuals to trace back to the beginning of the self and reality formation. This systematically fluctuating process of reality can result in both gains and losses. This dual effect could be better elucidated concerning the self-integration functions as an essential part of acquiring new knowledge in both physical and digital realities.

## Data Availability Statement

The datasets presented in this study can be found in online repositories. The names of the repository/repositories and accession number(s) can be found at: http://dx.doi.org/10.17632/6rcbcmnmyk.1.

## Ethics Statement

The study was conducted in accordance with the Helsinki Declaration (ethical allowance No. 6732 PTE/2017). The patients/participants provided their written informed consent to participate in this study.

## Author Contributions

JK and RHe: conceptualization and text edition. GV, IH, RHa, and IAT: data gathering. SR and AL: method control and statistical analysis. IT: text edition theoretical consideration. All authors contributed to the article and approved the submitted version.

## Conflict of Interest

The authors declare that the research was conducted in the absence of any commercial or financial relationships that could be construed as a potential conflict of interest.
