# Supplementary material for: Cognitive Gain or Handicap: Magical Ideation and Self-Absorption in Clinical and Non-clinical Participants
Source: Front Psychol. 2021 Feb 26;12:613074. doi: 10.3389/fpsyg.2021.613074 (PMC7952430; doi:10.3389/fpsyg.2021.613074)
Supplement: Supplementary file 1 [file Table_1.DOCX]

| Education in years in the entire sample:  8 class = 7.1 %; 11 class = 35.6 %; 12 class = 52 %; 17 and over = 5.3 %.  In sine morbo group:  8 class = 2.1 %; 11 class = 25.5 %; 12 class = 60.5 %; 17 and over = 1.6 %.  In schizophrenia spectrum disorder group:  8 class = 36 %; 11 class = 39 %; 12 class = 54 %; 17 and over = 4 %.  In anxiety disorder group:  8 class = 17.5 %; 11 class = 39.3 %; 12 class = 31.1 %; 17 and over = 12 %.  In mood disorder group:  8 class = 18.2 %; 11 class = 30 %; 12 class = 37 %; 17 and over = 18 %. |
| --- |

Supplement 1. The participant’s education is class years in the different groups of sample.
