# Supplementary material for: Cognitive Gain or Handicap: Magical Ideation and Self-Absorption in Clinical and Non-clinical Participants
Source: Front Psychol. 2021 Feb 26;12:613074. doi: 10.3389/fpsyg.2021.613074 (PMC7952430; doi:10.3389/fpsyg.2021.613074)
Supplement: Supplementary file 2 [file Table_2.DOCX]

| **Clinical A**  patients ANXIETY DISORDERS  F40.00-F48.00; N = 183 |  | **Clinical SSD** patients SCHIZOPHRENIA, SCHIZOTIPAL, DELUSIVE DISORDERS  F20.00-F29.00; N = 25 |
| --- | --- | --- |
|  |  |  |
| F41.20 (16.6), mixed anxiety with depression |  | F20.00 (25), schizophrenia |
| F41.10 (14.5), generalized  anxiety |  | F25.10 (20), schizoaffective disorder, depressive type |
| F41.80 (13.5), other specified anxiety disorder |  | F21.00 (15), schizotypal personality disorder |
| F43.20 (12.9), short depression reaction, unspecified |  | F23.80 (10), brief psychotic disorder  F22.90 (5), delusional disorder |
| F41.90 (12.4), unspecified anxiety disorder |  | F23.30 (5), brief psychotic disorder |
| F41.00 (11.8), panic disorder |  | F23.00 (5), other psychotic disorder |
| F43.22 (6.9), adjustment disorder, with anxiety F43.21 (3.8), adjustment disorders, with depressed mood |  | F23.10 (5), acute psychotic disorder, with schizophrenic symptoms  F20.60 (5), schizophrenia simplex  F20.30 (5) non-differentiated schizophrenia |
| F43.00 (2.2), acute stress disorder  F41.30 (2.2), other mixed anxiety disorder F45.10 (0.5), somatic symptom disorder |  |  |
|  |  | _______________________________________  **CLINICAL M** patients MOOD DISORDERS |
|  |  | F30.00-F39.00; N =110 MOOD DISORDERS |
|  |  |  |
|  |  | F33.20 (29.7), major depression with psychotic symptoms |
|  |  | F31.50 (24.3), bipolar I disorder, current or most recent episode depressed. With psychotic features |
|  |  | F32.20 (14.2), major depressive disorder, single episode. Severe |
|  |  | F32.10 (7.0), major depressive disorder, single episode. Moderate |
|  |  | F31.30 (4.3) bipolar I. disorder, current or most recent episode depressed. Mild |
|  |  | F31.40 (4.3), bipolar I. disorder, current or most recent episode depressed. Severe |
|  |  | F33.10 (3.4), bipolar I. disorder, current or most recent episode manic. Mild |
|  |  | F31.90 (2.7), bipolar I. disorder, current or most recent episode depressed. Unspecified |
|  |  | F31.80 (2.5), bipolar II. disorder |
|  |  | F33.01 (1.8), major depressive disorder, recurrent episode. Moderate |
|  |  | F33.30 (1.7), major depressive disorder, recurrent episode. With psychotic features |
|  |  | F32.30 (1.7), major depressive disorder, single episode. With psychotic features |
|  |  | F31.70 (0.9), bipolar I. disorder, current or most recent episode hypomanie. In partial remission |
|  |  | F32.50 major depression episode in full remission |
|  |  | F33.22 (0.9), major depressive disorder, recurrent episode. Severe |

Supplement 2. Characteristics and BNO - 10 classification codes for the clinical sample. Clinical A group contains patients with several anxiety disorders (N= 183), the Clinical SSD group involves patients with schizophrenia spectrum disorders (N = 25), and Clinical M mood disorder group consisted of 110 patients.
